# Genome-Wide Runs of Homozygosity Reveal Inbreeding Levels and Trait-Associated Candidate Genes in Diverse Sheep Breeds

**DOI:** 10.3390/genes16030316

**Published:** 2025-03-07

**Authors:** Rui Ma, Jiaxin Liu, Xiao Ma, Ji Yang

**Affiliations:** Frontiers Science Center for Molecular Design Breeding (MOE), State Key Laboratory of Animal Biotech Breeding, College of Animal Science and Technology, China Agricultural University, Beijing 100193, China; marui531@foxmail.com (R.M.); jiaxinliu2019@126.com (J.L.); mx20000326@163.com (X.M.)

**Keywords:** sheep, runs of homozygosity, inbreeding coefficient, candidate genes, fecundity, body size

## Abstract

Background: Quantifying and controlling the inbreeding level in livestock populations is crucial for the long-term sustainability of animal husbandry. However, the extent of inbreeding has not been fully understood in sheep populations on a global scale. Methods: Here, we analyzed high-depth genomes of 210 sheep from 20 worldwide breeds to identify the pattern and distribution of genome-wide runs of homozygosity (ROH) and detect candidate selected genes in ROH islands for agronomic and phenotypic traits. Results: Leveraging whole-genome sequencing data, we found a large number of short ROH (e.g., <1.0 Mb) in all breeds and observed the overall higher values of ROH statistics and inbreeding coefficient in European breeds than in Asian breeds and Dorper sheep. We identified some well-known candidate genes (e.g., *CAMK4*, *HOXA* gene family, *ALOX12*, *FGF11*, and *MTOR*) and 40 novel genes (e.g., *KLHL1*, *FGFRL1*, *WDR62*, *GDF6, KHDRBS2*, and *PAX1*) that are functionally associated with fecundity, body size, and wool-related traits in sheep. Based on the candidate genes, we revealed different genetic bases for the fecundity traits of European and Asian sheep. Conclusions: This study improves the resolution of ROH detection and provides new insights into genomic inbreeding and trait architecture in sheep as well as useful markers for future breeding practice.

## 1. Introduction

Sheep (*Ovis aries*) are one of the most important livestock, and they have provided daily necessities such as meat, wool, and milk to humans since the Neolithic Age. Following their domestication from Asiatic mouflon (*Ovis orientalis*) in the Fertile Crescent ~11,000 years ago [1,2], sheep have adapted to a variety of environments across the world and were subjected to human-implemented genetic improvements under different agroecosystems. During this process, sheep evolved diverse phenotypic (e.g., small body size) and agronomic (e.g., high fecundity) traits under long-term natural and artificial selection. Because only a few elite individuals with outstanding performance were selected to reproduce generation by generation, the selection scheme inevitably led to an increase in the inbreeding level in sheep populations. Inbreeding can impose an adverse effect on all sorts of traits, such as those related to fitness (e.g., fecundity), production (e.g., wool fineness), and morphology (e.g., body size) [3,4,5]. Also, inbreeding may cause a loss of genetic diversity and a decline of effective population size, which could be harmful to future breeding programs of sheep. In this context, assessing and controlling the inbreeding level is crucial for the long-term sustainability of the sheep industry. However, most of the previous genomic investigations of inbreeding in sheep only involved a single breed, one trait, or limited loci [6,7,8], leaving the extent of inbreeding not fully understood for multiple traits in different breeds on a global scale.

The conventional method for quantifying inbreeding level was based on pedigree-based data, but pedigree information is not always available and accurate in livestock populations [9,10]. With the development of genomic technologies, alternative methods based on SNP chip or genome-wide sequencing data have been employed to estimate inbreeding levels, among which a prevalent and efficient method is runs of homozygosity (ROH)-based inbreeding coefficient (F_ROH_) [11]. ROH are continuous homozygous segments inherited from identical haplotypes of parents and are common in human and animal populations [12,13]. Because inbreeding is one of the direct causes of the occurrence of ROH, ROH are considered as a suitable measure of inbreeding level [14]. In addition, the history of inbreeding can be inferred based on the length of ROH fragments. Long ROH fragments are usually produced by inbreeding of recent generations, as there are not enough generations for recombination to interrupt these ROH fragments. On the contrary, shorter ROH fragments are generated by inbreeding in history [8]. Recently, ROH detection throughout the whole genome has been widely used to evaluate genomic inbreeding (F_ROH_) of various livestock species, including sheep [8,15,16,17,18,19,20,21,22,23,24,25,26,27]. On the other hand, ROH are useful indicators for identifying selective signatures in the genome [28]. Genomic regions under strong artificial and natural selections can exhibit increased homozygosity around the target selected region [29,30], therefore causing the formation of ROH. ROH islands, which refer to the regions with high ROH frequency in a population, are frequently overlapped with genomic regions with selective signatures [29]. In this regard, ROH islands can be used to identify candidate genes related to important traits in livestock populations [31]. For example, Liu et al. [32] performed ROH detection among five Chinese sheep breeds with different tail types and identified candidate gene *PDGFD* in the ROH islands. Additionally, ROH also help to understand genetic diversity, population structure, and demographic history of livestock species [31].

**Figure 1:**
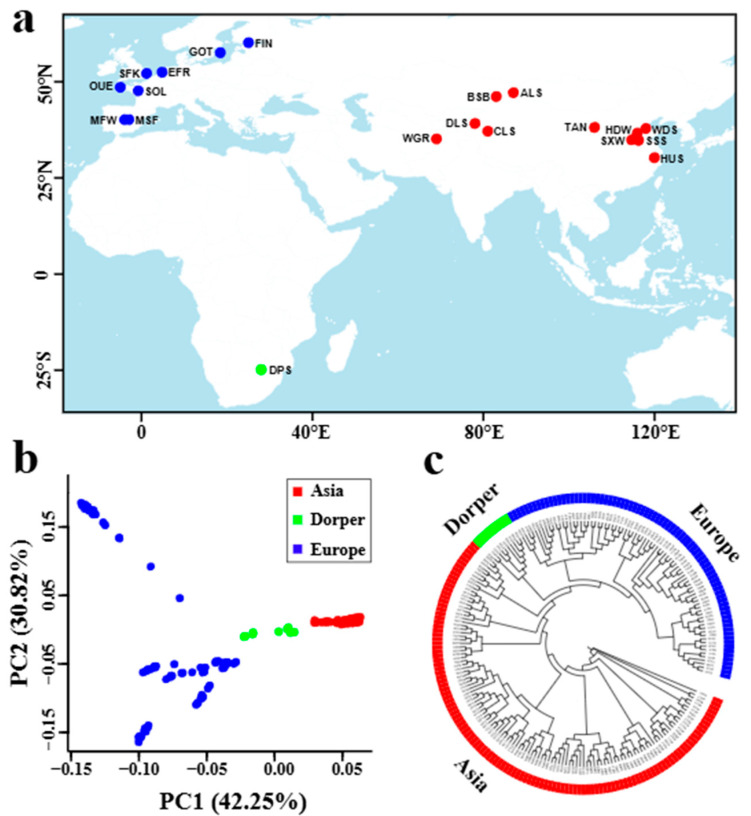
Breed origin and genetic structure of the studied sheep breeds from Asia (11 breeds) and Europe (8 breeds), along with the Dorper sheep from Africa. (**a**) The geographic distribution of the 20 sheep breeds. ALS, Altay; BSB, Bashibai; CLS, Cele Black; DLS, Duolang; DPS, Dorper; EFR, East Friesian Dairy; FIN, Finnsheep; GOT, Gotland; HDW, Large-tailed Han; HUS, Hu; MFW, Chinese Merino (fine wool); MSF, Chinese Merino (super-fine wool); OUE, Ouessant; SFK, Suffolk; SOL, Solognote; SSS, Sishui Fur; SXW, Small-tailed Han; TAN, Tan; WDS, Wadi; and WGR, Waggir. (**b**) Plots of principal components 1 and 2 for 210 individuals from the 20 breeds. (**c**) Maximum-likelihood tree construction for the 20 sheep breeds.

Here, we utilized whole-genome sequencing data of 20 sheep breeds (210 individuals) genetically originating from three continents (Figure 1a and Supplementary Table S1) to investigate the occurrence and distribution of genome-wide ROH in worldwide sheep populations. Based on the ROH, we aimed to (i) assess genetic status such as inbreeding coefficient, effective population size (Ne), and linkage disequilibrium (LD); (ii) identify candidate genes associated with phenotypic and agronomic traits in ROH islands. Particularly, we paid attention to previously under-studied traits (e.g., small body size) and compared genetic differences underlying the same trait (e.g., high fecundity) from sheep breeds in different continents. This study will improve our understanding of the genetic structure and demographic history of sheep breeds worldwide. Genes identified in ROH islands can be applied as genetic markers in future molecular breeding of sheep.

## 2. Materials and Methods

### 2.1. Whole-Genome Sequence Data

Whole-genome sequences of 210 sheep samples were obtained from our previous studies [33,34,35], including 11 breeds from Asia, 8 breeds from Europe, and the Dorper sheep from Africa according to their breed origin (i.e., genetic origin) (Figure 1a and Supplementary Table S1). In particular, the sheep samples represent breeds from different geographic regions of the world and with various morphological and production traits such as small body size, high fecundity, wool fineness, and dairy and meat production.

### 2.2. Read Alignment and SNP Calling

The raw sequences for each sample were ~83.93 Gb (76.43–109.87 Gb), with an average depth of 20.15× (16.73–28.01×) for clean reads (Supplementary Table S1). The raw Illumina reads were filtered to remove adapters and low-quality sequences using Trimmomatic (v0.36) [36] with parameters ‘SLIDINGWINDOW:4:15 MINLEN:50′. The clean reads were mapped to the sheep reference genome Oar_rambouillet_v1.0 (NCBI accession GCA_002742125.1) using the Burrows–Wheeler aligner (BWA-MEM) v.0.7.17-r1188 [37] with default parameters. Alignments were then transferred into BAM format via SAMtools v.1.11 [38], and duplicates were removed using GATK v.4.1.9.0 [39].

After mapping, SNP was called from the bam files by the HaplotypeCaller module implemented in GATK v.4.1.9.0 [39]. Raw GVCFs of individual samples were merged using the CombineGVCFs module and called for SNPs using the GenotypeGVCFs module. Subsequently, the candidate SNPs were identified by the SelectVariants module, and false-positive and nonbiallelic SNPs were filtered out via the VariantFiltering module with parameters “QUAL < 30.0‖QD < 2.0‖MQ < 40.0‖FS > 60.0‖SOR > 3.0‖ReadPosRankSum < −8.0”. In addition, SNPs with missing rates ≥ 0.1 and minor allele frequencies (MAF) < 0.05 were also filtered out from further analysis. After all the quality control procedures, a total of 29,468,844 SNPs remained in the downstream analysis.

### 2.3. Population Genetic Analysis

To investigate the population structure of the sheep samples used in this study, principal component analysis (PCA) was performed using PLINK v1.9 [40] based on three datasets (i.e., the whole samples, the Asian samples, and the European samples). Also, an approximately-maximum-likelihood phylogenetic tree was constructed for all samples using the FastTree v2.1.11 [41] with default parameters. The final tree was visualized via the online tool iTOL [42]. In addition, PopLDdecay v3.42 [43] was used to calculate linkage disequilibrium (LD) coefficients between all pairwise SNPs for each breed with the default parameter. LD decay was then plotted against the distance between two loci using the R program v4.1.0 [44]. To reveal the recent population demographic history of each breed, SNeP v1.1 [45] software was used to estimate the effective population size (Ne) of recent generations for each population with default parameters.

### 2.4. Identification of ROH

PLINK v1.9 [40] was used to identify runs of homozygosity (ROH) for each individual with a sliding window approach. The parameters defining the ROH were set as follows: (i) the minimum length of ROH was set to 500 kb; (ii) five missing SNPs and up to two possible heterozygous genotypes were allowed in a sliding window; (iii) the minimum number of SNPs that constituted the ROH was set to 100; (iv) the minimum SNP density in the ROH was set to one SNP every 50 kb; (v) the maximum gap between consecutive homozygous SNPs was 1000 kb; and (vi) the number of SNPs in a sliding window was set to 50. The average number (MN_ROH_) and average length (AL_ROH_) of ROH for each breed were calculated. The total length and total number of ROH for each individual were calculated, and the relationship between the two indicators was exhibited by a scatter plot. In order to better dissect the distribution of ROH among sheep breeds, we classified ROH into four categories as 0.5 Mb–1.0 Mb, 1.0 Mb–1.5 Mb, 1.5 Mb–2.0 Mb, and >2.0 Mb according to the length of the ROH. For each of the four categories, the mean sum of the ROH for each breed was calculated by summing all ROH in that category and dividing by the sample size of each breed. In addition, we also counted the number of ROH in each chromosome for each breed to reveal the difference in chromosomal distribution among breeds.

### 2.5. Calculation of Inbreeding Coefficient

The inbreeding coefficient based on ROH (F_ROH_) for each individual was calculated according to the following formula [46]:

F_ROH_ = L_ROH_/L_au_ (1)

where L_ROH_ is the total length of all the ROH in an individual, and L_au_ is the total length of the autosomal genome covered by SNPs (i.e., 2655.59 Mb in this study). A violin plot was drawn to reveal the difference in inbreeding levels among sheep breeds. Also, we used another measure (F_HOM_) to compute the genomic inbreeding coefficient based on the observed and expected number of homozygous genotypes using PLINK v1.9 [40]. Pearson’s correlation between F_ROH_ and F_HOM_ was estimated.

### 2.6. Detection of ROH Islands and Candidate Genes for Various Traits

To determine the ROH hotspots for each breed, the percentage of each SNP occurring in the ROH in each breed was calculated. For an SNP in a specific breed, the number of times this SNP was involved in the ROH was counted and then divided by the sample size of this breed. In this study, breeds with similar traits and from the same geographic region were combined to perform ROH hotspot analysis. Specifically, we identified ROH hotspots for the breeds with four different phenotypic or production traits: (i) high fecundity trait in Asian sheep using the merged data of Wadi sheep (WDS, *n* = 20), Hu sheep (HUS, *n* = 10), and Small-tailed Han sheep (SXW, *n* = 10); (ii) high fecundity trait in European sheep using the merged data of Finnsheep (FIN, *n* = 10) and Gotland sheep (GOT, *n* = 10); (iii) wool fineness trait based on the merged data of Chinese Merino sheep (fine wool) (MFW, *n* = 10) and Chinese Merino sheep (super fine wool) (MSF, *n* = 10); and (iv) small body size trait based on the data of Ouessant sheep (OUE, *n* = 10). The top 0.5% of the highest occurrence SNPs observed in the ROH in the breed/breeds with a particular trait was selected as the threshold for determining the ROH hotspots. A series of adjacent SNPs that exceeded this threshold were combined to form the ROH islands, which were visualized by plotting against the position of the SNPs along the chromosomes via a Manhattan plot. To identify the genes in the ROH islands and reveal their association with the corresponding trait, SNPs in the ROH islands were annotated according to the genomic annotation file of Oar_rambouillet_v1.0 (https://ftp.ncbi.nlm.nih.gov/genomes/all/GCF/002/742/125/GCF_002742125.1_Oar_rambouillet_v1.0/GCF_002742125.1_Oar_rambouillet_v1.0_genomic.gtf.gz, accessed on 2 January 2020). Functional enrichment analysis of candidate genes associated with the investigated traits in specific sheep breeds was conducted through the PANTHER v19.0 classification system [47]. The functions of the candidate genes in the ROH islands were also investigated using literature and the NCBI database.

## 3. Results

### 3.1. Population Structure

Using the 29,468,844 high-quality SNPs obtained from the genomes of 210 sheep samples worldwide (Figure 1a), we constructed a phylogenetic tree that divided the 20 studied sheep breeds into three subgroups of Asian, European, and Dorper sheep lineages (Figure 1c). Within each lineage, sheep samples from the same breed were well clustered into an independent branch, except that the samples from two Merino sheep populations (i.e., fine wool population and super fine wool population) were mixed and clustered together (Figure 1c). Principal component analysis (PCA) for all samples confirmed the geographic subdivision of Asian, European sheep, and Dorper sheep, and the samples of Asian sheep or Dorper sheep were clustered much more closely than that of European sheep (Figure 1b). PCA within Asian sheep and European sheep further revealed an overall obvious separation among different breeds, though the samples of several breeds (e.g., Altay sheep from Asia, super fine wool Merino sheep from Europe) were clustered loosely (Supplementary Figure S1).

### 3.2. Linkage Disequilibrium and Effective Population Size

**Figure 2:**
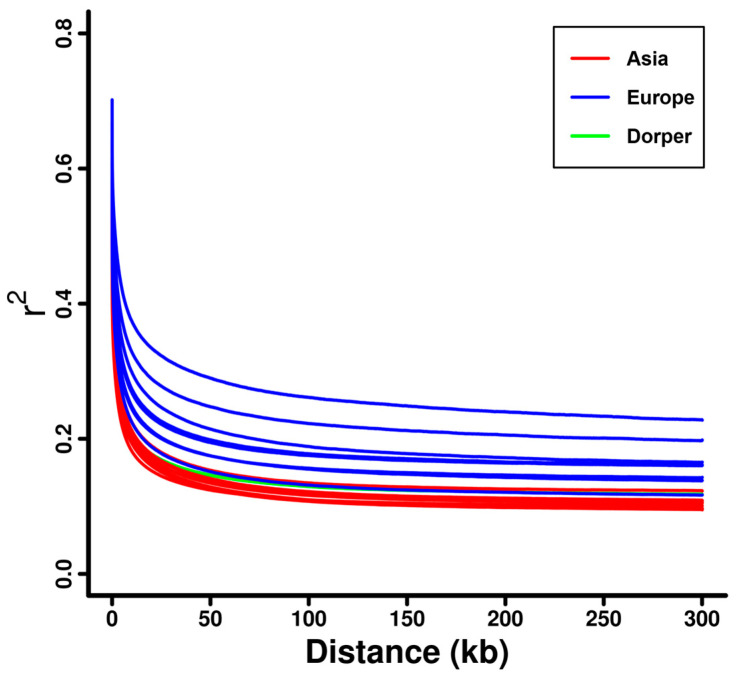
LD decay for the sheep breeds from Asia and Europe, as well as the Dorper sheep (an African breed).

**Figure 3:**
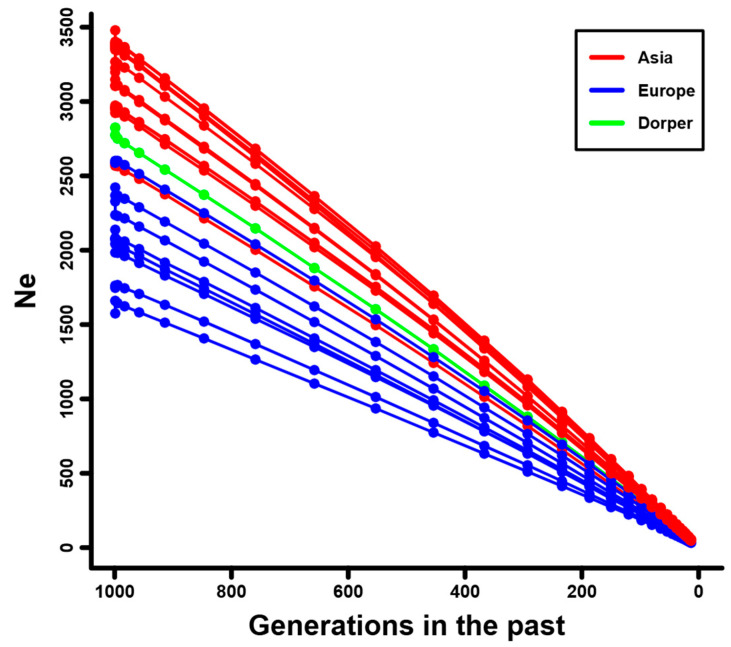
Estimated effective population sizes (Ne) for the sheep breeds from Asia and Europe, as well as the Dorper sheep (an African breed). The Ne is the historical effective population size of the studied sheep breeds or their ancestral populations.

The decay of linkage disequilibrium (LD) against genomic distance among the 20 domestic sheep breeds showed that European sheep (e.g., Ouessant, Solognote, and Gotland sheep) had a higher level of LD than that in Dorper sheep and Asian sheep (e.g., Hu, Wadi, and Small-tailed Han sheep) (Figure 2 and Supplementary Figure S2). For individual breeds, Ouessant sheep had the highest level of LD, followed by Solognote and Gotland sheep (Supplementary Figure S2). The estimated Ne for the domestic sheep breeds in the past 1000 generations exhibited a similar trend of decline over time (Supplementary Figure S3). At specific generations tested (e.g., 1000 generations ago), Ne estimates were obviously larger in Asian sheep (e.g., Hu, Wadi, and Small-tailed Han sheep) than that in Dorper sheep and European sheep (e.g., Ouessant, Solognote, and Gotland sheep) (Figure 3 and Supplementary Figure S3). This pattern was inversely correlated with the pattern of LD level in the domestic sheep breeds. Ouessant, Solognote, and Gotland sheep had the smallest Ne (Supplementary Figure S3).

### 3.3. Genomic Characterization of ROH

To characterize genome-wide ROH in the studied sheep breeds, we first examined the average number of total ROH and the average length per ROH for each sheep breed. As shown in Table 1, the average ROH numbers ranged from 41.5 in Small-tailed Han sheep to 559.5 in Ouessant sheep. The top three highest average ROH numbers were found in three European breeds, Ouessant (559.5), Solognote (377.6), and Gotland sheep (293.8), and the first three lowest average ROH numbers were observed in three Asian breeds, Small-tailed Han (41.5), Altay (42.3), and Bashibai sheep (45.9) (Table 1). Regarding the average length per ROH, the pattern among the studied sheep breeds was similar to the average number of total ROH. The highest and lowest values of the average ROH length were still found in European breeds (e.g., 0.909 Mb in East Friesian Dairy sheep, 0.86 Mb in Ouessant sheep, and 0.847 Mb in Solognote sheep) and Asian breeds (e.g., 0.708 Mb in Hu sheep, 0.762 Mb in Small-tailed Han sheep, and 0.764 Mb in Large-tailed Han sheep), respectively (Table 1).

**Figure 4:**
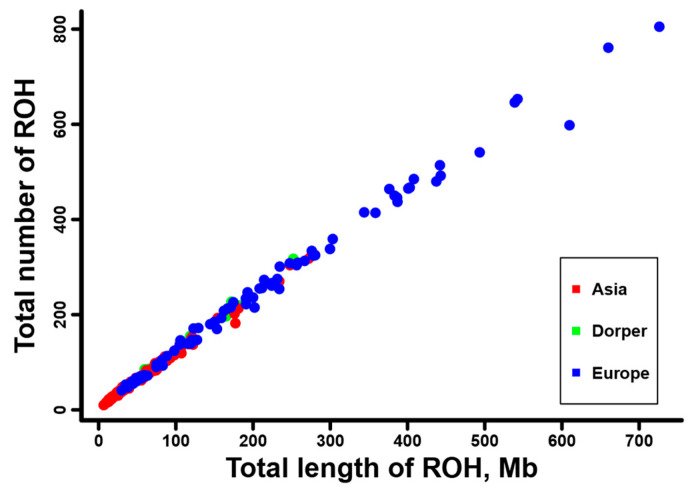
The total number and total length of ROH per individual for the sheep breeds from Asia and Europe, as well as the Dorper sheep (an African breed).

**Figure 5:**
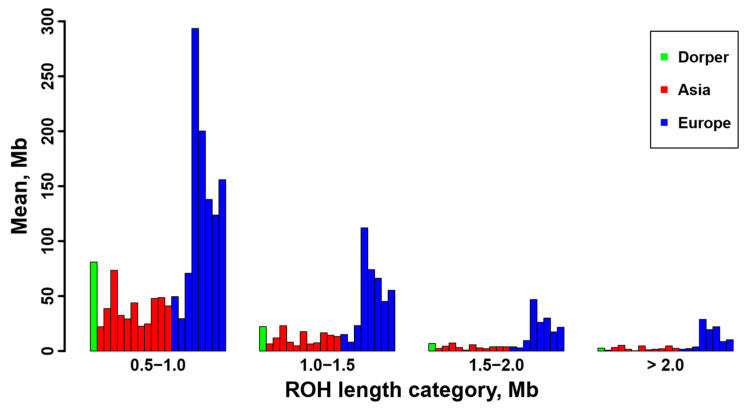
The mean sum of ROH length per individual for the sheep breeds from Asia and Europe, as well as the Dorper sheep (an African breed).

**Figure 6:**
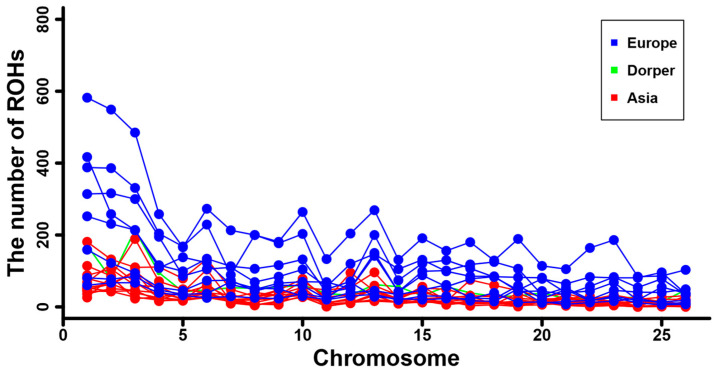
The number of ROH per chromosome for the sheep breeds from Asia and Europe, as well as the Dorper sheep (an African breed).

Furthermore, we calculated the total number and total length of the ROH for each individual, and they are displayed in Supplementary Figure S4. A positive and strong correlation was observed between the total ROH number and the total ROH length across individuals. Consistent with the results at breed level (Table 1), sheep individuals from Europe (e.g., Ouessant, East Friesian Dairy, Gotland, and Solognote individuals) exhibited the highest values of both the total number and total length of ROH (Figure 4 and Supplementary Figure S4). As for the mean sum of the ROH in the four length categories, short ROH segments of 0.5–1.0 Mb accounted for the largest percentage (53.8–83.5%) of the whole ROH length in all breeds, followed by segments of 1.0–1.5 Mb (13.4–25.9%), 1.5–2.0 Mb (2.4–11.7%), and greater than 2.0 Mb (0.6–8%) (Figure 5). European breeds had higher values of the mean sum of the ROH than Asian breeds and Dorper sheep in all length categories, and Ouessant sheep from Europe showed the highest mean sum values (Figure 5 and Supplementary Figure S5). Regarding the relationship between ROH segment length and generations of inbreeding events, the expected length of ROH theoretically follows an exponential distribution with a mean equal to 1/2 g Morgans, where g is the number of generations since the common ancestor. Based on this formula, ROH segments of 0.5–1.0 Mb, 1.0–1.5 Mb, 1.5–2.0 Mb, and >2.0 Mb correspond to 100–50 generations, 50–33.3 generations, 33.3–25 generations, and <25 generations of inbreeding events, respectively. In addition, we found a similar trend for the number of ROH among different chromosomes across breeds (Supplementary Figure S6). Generally, the number of ROH per chromosome tended to reduce with the decrease of chromosomal length. In most breeds, the highest and lowest number of ROH were detected on chromosome 1, and the highest percentage of total ROH length relative to chromosomal size was observed on chromosome 2 (Figure 6).

### 3.4. Inbreeding Coefficient Based on ROH

**Figure 7:**
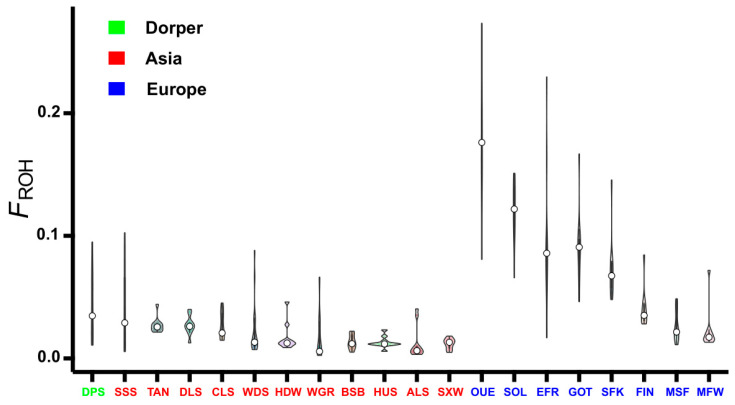
Distribution of inbreeding coefficient F_ROH_ in the 20 sheep breeds. ALS, Altay; BSB, Bashibai; CLS, Cele Black; DLS, Duolang; DPS, Dorper; EFR, East Friesian Dairy; FIN, Finnsheep; GOT, Gotland; HDW, Large-tailed Han; HUS, Hu; MFW, Chinese Merino (fine wool); MSF, Chinese Merino (super-fine wool); OUE, Ouessant; SFK, Suffolk; SOL, Solognote; SSS, Sishui Fur; SXW, Small-tailed Han; TAN, Tan; WDS, Wadi; and WGR, Waggir.

Based on the total length of the ROH, the inbreeding coefficient F_ROH_ was estimated for each individual, and then the mean value of F_ROH_ was calculated for each sheep breed. Overall, the F_ROH_ values varied obviously among different breeds and different geographic regions (Figure 7 and Table 1). For example, most European breeds had a significantly higher level of F_ROH_ than Asian breeds and Dorper sheep. The top three highest values of F_ROH_ were found in Ouessant (0.1812), Solognote (0.1204), and East Friesian Dairy sheep (0.0965) from Europe, while the first three lowest values were detected in Small-tailed Han (0.0119), Altay (0.0124), and Hu sheep (0.0131) from Asia.

The other estimated inbreeding coefficient, F_HOM_, generally exhibited higher values than F_ROH_ in almost all sheep breeds except Bashibai sheep and fine wool Merino sheep (Table 1). The F_HOM_ value was only negative in Bashibai sheep and ranged from −0.0013 in Bashibai sheep to 0.3467 in Ouessant sheep (Table 1). The Pearson’s correlations between F_ROH_ and F_HOM_ in the studied sheep breeds were from 0.595 in Solognote sheep to 0.995 in Sishui Fur sheep, and the average correlation coefficient across all breeds was 0.952 (Table 1).

### 3.5. ROH Islands and Candidate Genes for Various Traits

**Figure 8:**
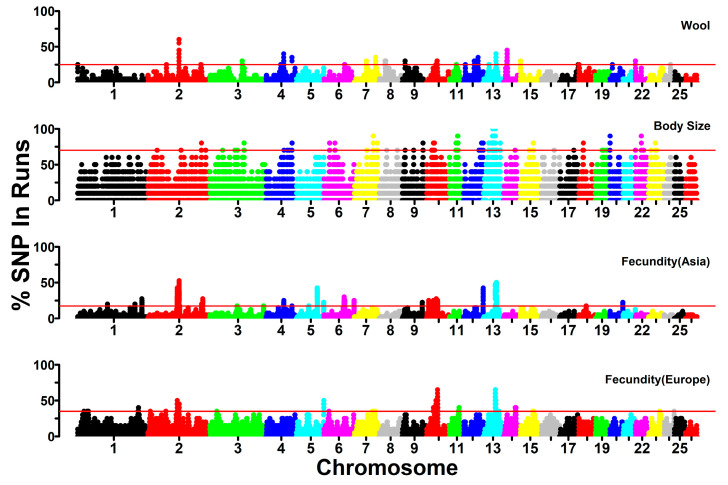
Manhattan plot of SNP occurrence within ROH regions along the chromosome for the investigated traits. The horizontal red line represents the threshold to define the ROH islands.

To identify genomic regions subjected to selection from the perspective of ROH, we examined the ROH islands in the sheep populations with the traits of high fecundity, wool fineness, and small body size (see Section 2.6). Particularly, we separately investigated the high fecundity trait of European and Asian sheep, which were under different livestock production systems. We estimated the occurrence of SNPs in the ROH and selected the top 0.5% of the highest occurrence SNPs as the threshold of the ROH islands for each trait. The ROH islands were illustrated through Manhattan plots of SNP occurrence against SNP locations along the chromosome (Figure 8). In total, we identified 18, 33, 27, and 42 ROH islands (14, 25, 20, and 32 islands containing annotated protein-coding genes) for the high fecundity trait of European sheep and Asian sheep, wool fineness trait, and small body size trait, respectively. The different number and different chromosomal distribution of ROH islands among the investigated traits may reveal distinct breeding history and selective pressure of each trait in the studied breeds. Detailed information about the identified ROH islands, such as the genomic location of ROH islands and the number of SNPs and candidate genes within ROH islands, is shown in Table 2.

Through the annotation of the SNPs in the ROH islands according to the genomic annotation file of Oar_rambouillet_v1.0 (NCBI accession GCF_002742125.1), we obtained 79 and 211 candidate genes for the fecundity trait of European sheep and Asian sheep, 174 candidate genes for the wool-related trait, and 325 candidate genes for the body size trait (Tables S2–S5). Notably, the functions of some candidate genes for the fecundity trait (e.g., *CAMK4*, *KLHL1*, *WDR36*, *LTBP4*, *EGLN2*, *CAPNS1*, *WDR62*, *ATRN*, *HSPA12B, SPEF1*, *CENPB*, *CDC25B*, *SMOX*, *AGTR1*, *CPA3*, and *ZNF146* in European sheep; *CAMK4*, *KLHL1*, *WDR36*, *FGFRL1*, *RNF212*, *HOXA* gene family, *EVX1*, *TTLL4*, *PLCD4*, *SLC11A1*, *AAMP*, *SPAG16*, *FSIP2*, *ABHD16B*, *NPBWR2*, *MYT1*, *GNRH2*, *OXT*, *AVP*, *RNPEP*, *PTPN7*, *CRYL1*, *IFT88*, *IL17D*, *STAG1*, *ADCY10*, *CXCR1*, *CXCR2*, and *PHLDA3* in Asian sheep) have been reported to be associated with reproduction, such as oocyte maturation, embryo development, spermatogenesis, pregnancy, litter size, and ovarian disease [48,49,50,51,52,53,54,55,56]. Most of the fecundity-associated candidate genes in European sheep (59 of 79) and Asian sheep (191 of 211) were different, probably indicating distinct genetic bases for the fecundity trait in the two sheep groups from different agroecosystems. Moreover, we found that candidate genes for the wool-related trait (e.g., *SHCBP1*, *HOXA10*, *MTOR*, *MC1R*, and *TCF25*) were functionally involved in hair follicle induction, morphogenesis and cycling, the formation of white wool, apoptosis of hair follicle stem cells, and coat color [57,58,59,60,61], while candidate genes for the body size trait (e.g., *GDF6*, *KHDRBS2*, *PAX1*, *PTPN7*, *ALOX12*, *FGF11*, *TP53*, *KDM6B*, *CHD3*, *HES7*, *RPL26*, *PIK3R6*, *PIK3R5*, *NTN1*, and *TOM1L2*) were functionally relevant to skeletal patterning, small or large body size, physical morphology, and tall stature phenotype [62,63,64,65,66,67]. Functional enrichment analysis of the candidate genes in Panther revealed significant (*p* < 0.05) and biologically important GO terms and pathways for investigated traits (Supplementary Table S6). For instance, two biological process GO terms (Reproductive process and Reproductive structure development) and one pathway (Vasopressin synthesis) were associated with the fecundity trait of Asian sheep.

## 4. Discussion

We examined genome-wide ROH patterns in worldwide sheep populations using high-depth sequencing data (i.e., an average sequencing depth of ~20.15×) of 210 sheep from 20 diverse sheep breeds. Compared to SNP chips, the use of whole-genome sequencing data can cover the entire genome and achieve greater resolution and accuracy for ROH detection, and subsequently provide a deeper understanding of genomic inbreeding and trait-associated candidate selected genes [12,31].

The results from the phylogenetic tree and PCA consistently showed that sheep breeds were largely classified into clusters according to their genetic origin (i.e., Asia, Europe, and Africa) and breed attribution (Figure 1b,c and Supplementary Figure S1), which was in line with previous findings on sheep population structure [33,35]. This could reflect the reliability of the genomic data of investigated sheep individuals. The linkage disequilibrium (LD) decay and effective population size (Ne) analysis revealed distinct demographic histories among the 20 sheep breeds. European breeds, such as Ouessant, Solognote, and Gotland sheep, exhibited higher levels of LD (e.g., higher r^2^ values), while Asian breeds and Dorper sheep displayed lower levels of LD (e.g., lower r^2^ values) (Figure 2 and Supplementary Figure S2). This finding was congruent with recent studies investigating sheep breeds from different continents [35], and may be attributed to small effective population sizes (Figure 3 and Supplementary Figure S3) [68] and extensive breeding practices in European sheep populations [69].

We found a non-uniform distribution of ROH among different sheep breeds. The average number of total ROH was 143.81, ranging from 41.5 ± 13.54 in Small-tailed Han sheep from Asia to 559.5 ± 159.06 in Ouessant sheep from Europe (Table 1). This quantity of ROH was much higher than previous reports in sheep (e.g., 23.8 ± 13.8) [8] and other livestock (e.g., cattle, 82.3 ± 9.83) [70] based on SNP chip data. With regard to different ROH length categories, a vast majority of the ROH belonged to short ROH segments of 0.5–1.0 Mb (53.8–83.5%) and 1.0–1.5 Mb (13.4–25.9%) (Figure 5). Compared to former studies, which detected a considerable number of long ROH (e.g., >4.0 Mb) from SNP chips [8,32], our high-depth whole-genome sequencing data greatly improved the resolution of short ROH, especially for those shorter than 1.0 Mb. The above results illustrate the advantage of whole-genome sequencing data for accurate and efficient identification of genomic ROH. Despite the aforementioned differences in detailed ROH statistics between this study and previous SNP chip-based research, our results revealed a similar ROH pattern for sheep breeds from different geographic regions. European breeds showed an overall higher ROH number and higher mean sum of ROH length than Asian breeds and Dorper sheep in all ROH length categories (Figure 4 and Figure 5, Supplementary Figures S4 and S5), indicating a high level of inbreeding in European populations [8]. In addition, the chromosomal distribution of ROH (Figure 6 and Supplementary Figure S6) was in agreement with previous studies that the number of ROH and the percentage of ROH length relative to chromosomal size are correlated with the chromosome length [71,72].

The F_ROH_ ranged from 0.0119 in Small-tailed Han sheep from Asia to 0.1812 in Ouessant sheep from Europe, and the level of F_ROH_ was significantly higher in European breeds than those in Asian breeds and Dorper sheep (Figure 7 and Table 1). These results were similar to previous sheep studies [8,32] and implied that Asian sheep and Dorper sheep populations are less inbred and possess higher levels of genetic diversity as compared to European sheep populations. We also estimated another genomic inbreeding coefficient, F_HOM_, which revealed analogous inbreeding patterns among the 20 sheep breeds and had a high correlation with F_ROH_. This indicated the reliability of F_ROH_ as an effective measure of the inbreeding coefficient. Interestingly, Ouessant sheep had the highest values in ROH statistics, inbreeding coefficient, and LD level, as well as the smallest Ne. Ouessant sheep are famous for their extremely small size and are mainly distributed on Ouessant island, separated from the European continent [73]. The isolated environments restrict the genetic exchange between OUE sheep and other European sheep breeds [74], thus leading to a specific breeding history and genetic characteristics for this breed.

We identified 79 genes in 18 ROH islands, 211 genes in 33 ROH islands, 174 genes in 27 ROH islands, and 325 genes in 42 ROH islands across the sheep genome in corresponding breeds for the fecundity trait of European sheep and Asian sheep, wool-related trait, and body size trait, respectively (Table 2). Regarding the fecundity trait, we observed a large proportion of different candidate genes for Asian (191 out of 211 genes) and European prolificacy sheep (59 out of 79 genes), indicating a potential distinct genetic basis underlying their high fertility [75]. Among the candidate genes, we detected some important genes (e.g., *CAMK4*, *HOXA* gene family, *GNRH2*, *FSIP2*, and *CRYL1*), which have been reported to be associated with reproduction in sheep. For example, *CAMK4* was found to be related to reproduction in Hu sheep [48] and the main factor regulating the reproductive behavior in Angus cattle [76]. The *HOXA* gene family (e.g., *HOXA1-HOXA5*, *HOXA9-HOXA11*, and *HOXA13)* has been linked to fertility in Hu sheep [55] and can enhance myometrial cell contractility and regulate female reproductive tract development in humans [77,78] and control cell differentiation and morphogenesis during mouse embryonic development [79,80]. *GNRH2* has been documented to be associated with fertility in Hu sheep [55]. *FSIP2* is a potential biomarker to assess the quality of frozen–thawed ram sperm [54]. *CRYL1* is associated with sexual behavior in Rasa Aragonesa rams [81] and is a useful diagnostic target for intrauterine growth restriction in sheep [82]. Notably, we revealed 30 novel candidate genes (*KLHL1*, *WDR36, FGFRL1*, *RNF212*, *EVX1*, *TTLL4*, *PLCD4*, *AAMP*, *SPAG16*, *ABHD16B*, *NPBWR2*, *RNPEP*, *PTPN7*, *IL17D*, *STAG1*, *ADCY10*, *CXCR1*, *CXCR2*, and *PHLDA3* in Asian sheep; *KLHL1*, *WDR36*, *LTBP4*, *EGLN2*, *CAPNS1*, *WDR62*, *ATRN*, *HSPA12B*, *SPEF1*, *CDC25B*, *AGTR1*, *CPA3*, and *ZNF146* in European sheep) which were reported to be related to reproduction in humans or other animals but previously not known to associate with the sheep fecundity trait. For instance, *KLHL1* is involved in oocyte maturation in humans [49]. *FGFRL1* is related to the prolificacy trait in goats [53]. The expression of *PHLDA3* in ovarian cortical tissues is associated with primary ovarian insufficiency in humans [56]. *LTBP4* can regulate stromal and thecal cells during bovine ovarian development [50]. *WDR62* is required for both oocyte meiotic maturation and spermatogenesis in mice [83,84]. The interaction of *CDC25B* with *YWHAH* can affect mouse oogenesis and oocyte maturation [85]. *ZNF146* can regulate cell cycle progression in human ovarian cancer cells [52]. For the body size trait, we discovered 5 previously known (*ALOX12*, *FGF11*, *RPL26*, *PIK3R5*, and *NTN1*) [64,65,86] and 10 novel candidate genes (*GDF6*, *KHDRBS2*, *PAX1*, *PTPN7*, *TP53*, *KDM6B*, *CHD3*, *HES7*, *PIK3R6*, and *TOM1L2*) associated with sheep physical morphology and stature. Of the novel genes, *PTPN7* can affect height in infancy and early childhood in humans [63]. *GDF6* can determine multiple joint and skeletal patterning [62], and *PAX1* can affect facial and other physical morphology [87] in mice. *KDM6B* and *CHD3* were found to be associated with body size traits in pigs [66]. *PIK3R6* showed direct functional associations with body size and height in cattle [67]. For wool-related traits, we identified several famous genes that have been well-known to control proliferation and apoptosis of hair follicle stem cells (*MTOR*) [60], wool quality (*SHCBP1*) [61], and wool color (*HOXA10*, *MC1R*, and *TCF25*) [57,58,59] in sheep.

Apart from the above findings, the present study has potential limitations. First, this study did not include information about the gender of the sheep individuals. Thus, the sex-based difference in ROH statistics and inbreeding levels cannot be addressed. Second, the sample size of each sheep breed is relatively small. As sampling may affect the results of ROH, LD, and Ne, future studies with a larger sample size are needed to verify the findings here and further enhance our understanding of ROH and genetic diversity in sheep populations. Third, the genomic inbreeding was analyzed using F_ROH_ and F_HOM_ but not compared with additional measures such as those based on identical by descent (IBD) segment [88,89].

## 5. Conclusions

In conclusion, we used high-depth whole-genome sequencing data to reveal genome-wide ROH patterns, genomic breeding, and the genetic architecture of various traits in 20 sheep breeds. We found that most European breeds (six of eight breeds) have a larger total number and a longer total length of ROH, higher inbreeding levels, and smaller effective population sizes than Asian breeds. Notably, our results significantly improved the resolution of ROH detection by discerning much shorter ROH less than 1 Mb, which account for 53.8–83.5% of total ROH in different breeds. Based on ROH islands, we identified 270, 325, and 174 candidate genes associated with fecundity, body size, and wool-related traits. We also disclosed a large majority of different candidate genes between European (59 of 79 genes, 74.7%) and Asian (191 of 211 genes, 90.5%) prolificacy sheep, indicating a potentially different genetic basis for their fecundity traits. Importantly, we discovered 30 and 10 novel genes responsible for sheep fecundity and body size traits, respectively. Our study contributes to a deeper understanding of sheep population genetics and provides valuable new markers for future molecular breeding.

## Figures and Tables

**Table 1:** Summary statistics for ROH and genomic inbreeding coefficients.

Breeds	ROH Number (Mean ± SD)	ROH Length, Mb (Mean ± SD)	F_ROH_ (Mean ± SD)	F_HOM_ (Mean ± SD)	r(F_ROH_ − F_HOM_)
DPS	143.80 ± 93.37	0.784 ± 0.297	0.0425 ± 0.0288	0.0730 ± 0.0523	0.913
SXW	41.50 ± 13.54	0.762 ± 0.318	0.0119 ± 0.0043	0.0271 ± 0.0148	0.724
WDS	71.65 ± 60.10	0.809 ± 0.379	0.0218 ± 0.0209	0.0539 ± 0.0350	0.922
SSS	135.20 ± 116.95	0.805 ± 0.356	0.0410 ± 0.0378	0.0849 ± 0.0698	0.995
HDW	59.00 ± 32.74	0.764 ± 0.322	0.0170 ± 0.0114	0.0411 ± 0.0181	0.928
HUS	49.30 ± 15.87	0.708 ± 0.232	0.0131 ± 0.0047	0.0469 ± 0.0091	0.718
TAN	85.30 ± 19.71	0.843 ± 0.387	0.0271 ± 0.0065	0.0489 ± 0.0111	0.893
ALS	42.30 ± 38.79	0.779 ± 0.332	0.0124 ± 0.0135	0.0154 ± 0.0250	0.985
BSB	45.90 ± 17.74	0.785 ± 0.335	0.0136 ± 0.0059	-0.0013 ± 0.0319	0.837
CLS	88.00 ± 35.69	0.798 ± 0.309	0.0264 ± 0.0113	0.0518 ± 0.0229	0.988
DLS	88.20 ± 22.35	0.810 ± 0.357	0.0269 ± 0.0077	0.0407 ± 0.0120	0.952
MFW	75.80 ± 56.17	0.798 ± 0.334	0.0228 ± 0.0175	0.0160 ± 0.0435	0.979
MSF	90.40 ± 44.41	0.773 ± 0.294	0.0263 ± 0.0127	0.0285 ± 0.0381	0.956
WGR	54.00 ± 61.41	0.789 ± 0.351	0.0161 ± 0.0205	0.0355 ± 0.0488	0.934
FIN	130.50 ± 50.17	0.821 ± 0.354	0.0403 ± 0.0167	0.0856 ± 0.0305	0.971
OUE	559.50 ± 159.06	0.860 ± 0.392	0.1812 ± 0.0552	0.3467 ± 0.0689	0.713
SOL	377.60 ± 75.30	0.847 ± 0.385	0.1204 ± 0.0257	0.2167 ± 0.0258	0.595
EFR	281.90 ± 155.49	0.909 ± 0.449	0.0965 ± 0.0599	0.1517 ± 0.0785	0.937
SFK	234.80 ± 81.66	0.830 ± 0.355	0.0734 ± 0.0275	0.1350 ± 0.0503	0.956
GOT	293.80 ± 85.05	0.827 ± 0.358	0.0915 ± 0.0318	0.2005 ± 0.0460	0.817
all	143.81 ± 149.00	0.830 ± 0.371	0.0450 ± 0.0495	0.0835 ± 0.0914	0.952
*p*-value	2 × 10^−16^	3.23 × 10^−14^	2 × 10^−16^	2 × 10^−16^	/

Note: The significance of differences in ROH statistics between sheep breeds was tested using multivariate analysis of variance in R v4.1.0 [44]. ALS, Altay; BSB, Bashibai; CLS, Cele Black; DLS, Duolang; DPS, Dorper; EFR, East Friesian Dairy; FIN, Finnsheep; GOT, Gotland; HDW, Large-tailed Han; HUS, Hu; MFW, Chinese Merino (fine wool); MSF, Chinese Merino (super-fine wool); OUE, Ouessant; SFK, Suffolk; SOL, Solognote; SSS, Sishui Fur; SXW, Small-tailed Han; TAN, Tan; WDS, Wadi; and WGR, Waggir.

**Table 2:** List of ROH islands identified in the studied breeds with different traits.

Traits	Chromosome	Number of SNPs	Start (bp)	End (bp)	Number of Genes
Fecundity (European breeds)	1	3568	260,516,400	261,008,501	3
	2	7869	125,362,089	125,929,311	2
	2	4788	133,972,981	134,262,353	0
	2	258	134,368,535	134,393,056	0
	5	1810	116,823,462	117,482,846	3
	10	1405	28,356,529	28,915,246	6
	10	3414	39,546,231	40,127,304	1
	10	7324	40,690,153	41,457,031	2
	10	4390	45,284,988	45,892,572	0
	10	125	45,893,708	45,917,433	0
	10	11,555	46,066,651	47,479,008	3
	11	903	41,691,551	41,766,808	4
	13	1736	51,484,744	52,073,593	3
	13	966	53,029,769	53,185,643	1
	13	3722	53,185,987	53,789,155	14
	13	583	53,789,304	53,954,964	2
	14	4564	49,339,955	49,705,671	22
	14	2307	52,590,703	52,871,923	14
Fecundity (Asian breeds)	1	1264	127,784,437	128,112,826	5
	1	1409	245,349,451	245,668,687	0
	1	4564	275,364,067	276,000,826	6
	2	12,148	125,310,139	126,205,892	3
	2	13,140	133,294,917	134,500,712	4
	2	476	230,125,601	230,160,780	1
	2	2246	234,992,972	235,522,246	24
	4	2492	75,493,738	75,983,090	17
	5	5524	88,822,803	89,607,365	2
	5	1030	116,948,615	117,463,164	3
	6	19,452	86,306,956	87,351,718	1
	6	481	87,513,615	87,568,336	0
	6	265	87,568,659	87,587,663	0
	6	4559	88,034,854	88,359,440	1
	6	3883	89,450,099	90,010,975	0
	6	2309	129,274,460	129,779,428	20
	9	2850	85,450,258	86,050,942	4
	10	9390	8,565,265	9,194,043	1
	10	1349	28,371,801	28,939,707	6
	10	516	37,585,363	37,879,321	8
	10	223	37,884,095	37,921,937	2
	10	3402	39,546,881	40,127,304	1
	10	5702	40,239,681	41,017,766	2
	10	1980	43,965,011	44,184,394	0
	10	846	44,247,494	44,336,764	0
	10	3548	45,367,439	45,879,518	0
	10	3979	46,456,617	46,999,992	2
	10	656	47,185,534	47,220,162	0
	12	2981	83,917,395	84,495,501	20
	13	2951	51,181,396	52,061,687	3
	13	1826	54,104,798	54,613,939	28
	13	2676	55,938,389	56,804,834	41
	20	1692	54,679,439	55,249,641	6
Wool	2	7568	133,087,142	133,777,850	5
	3	4451	138,495,186	138,938,836	4
	3	287	138,939,171	138,994,723	0
	3	1226	138,995,024	139,160,139	3
	4	2784	75,441,569	75,983,090	18
	4	4650	110,595,183	111,105,973	3
	7	2608	52,547,867	53,015,509	7
	7	2653	91,870,692	92,062,931	3
	7	4261	92,063,098	92,595,740	16
	8	9502	24,759,801	25,746,266	4
	8	4306	26,532,538	27,109,398	0
	8	357	27,973,982	28,000,808	0
	9	7038	11,737,256	12,244,991	0
	10	17	44,162,922	44,165,123	0
	10	683	45,412,636	45,501,889	0
	12	3758	45,584,092	46,097,353	10
	12	184	55,301,863	55,390,658	3
	12	4656	61,885,513	62,419,297	2
	13	1862	54,104,588	54,637,805	28
	14	1080	13,410,344	13,668,206	1
	14	2632	13,894,458	14,142,159	7
	14	5094	14,142,386	15,078,390	23
	14	9	15,078,540	15,079,377	0
	14	3030	15,082,920	15,649,580	24
	14	491	15,652,185	15,715,741	1
	15	5113	1,112,183	1,642,914	9
	22	4365	220,584	750,448	5
Body size	2	1254	229,295,362	229,454,903	1
	3	2332	146,197,096	146,567,912	12
	3	343	146,568,243	146,632,643	3
	3	992	146,714,711	146,842,536	5
	3	166	146,892,649	146,927,816	1
	3	368	146,927,959	146,985,136	3
	4	2033	111,126,770	111,331,638	4
	6	3612	24,489,901	24,762,969	0
	6	2576	47,322,449	47,501,103	0
	7	7033	81,665,952	82,295,710	2
	7	16	99,468,631	99,556,337	0
	7	1613	99,603,078	99,736,702	0
	9	4933	88,768,178	89,181,444	6
	10	432	28,371,264	28,503,446	3
	10	3005	33,282,855	33,815,588	7
	11	2919	28,211,580	28,765,976	14
	11	4502	34,884,456	35,764,740	55
	11	766	35,764,816	36,145,259	31
	11	667	36,145,459	36,464,031	32
	11	592	36,464,533	36,602,225	11
	11	220	37,050,098	37,095,273	1
	12	8452	76,867,182	77,507,534	11
	12	4075	83,728,664	84,298,251	18
	13	1209	23,661,787	23,938,741	2
	13	5489	36,077,863	36,646,913	6
	13	6143	39,564,442	40,266,374	19
	13	6582	40,266,727	40,995,074	10
	13	2763	40,995,703	41,369,024	3
	13	279	41,582,319	41,607,838	0
	13	3826	41,608,135	42,271,886	8
	13	724	45,752,803	45,820,785	0
	13	2867	47,100,995	47,377,999	0
	13	2406	47,378,390	47,619,133	0
	13	7034	47,619,182	48,644,663	14
	13	1917	51,441,819	52,093,035	3
	13	14	52,093,271	52,095,647	0
	13	1936	72,541,472	72,890,366	12
	15	3608	55,819,107	56,435,858	11
	18	464	19,405,282	19,474,356	0
	20	3362	98,697	646,780	4
	22	2014	25,467,538	25,974,815	18
	23	997	30,189,190	30,308,004	2

## Data Availability

The whole genome re-sequence data used for the study are publicly available under the project numbers listed in Table S1. All scripts used for this work were performed using open-source software tools and are available from the corresponding authors upon request.

## Supplementary Materials

The following supporting information can be downloaded at https://www.mdpi.com/article/10.3390/genes16030316/s1, Figure S1. Principal component analysis for Asian and European sheep breeds. (a) Principal component analysis for Asian sheep breeds. (b) Principal component analysis for European sheep breeds; Figure S2. LD decay across genomic distance in the 20 sheep breeds; Figure S3. Estimates of Ne for the 20 sheep breeds (or their ancestral populations) from 1000 years ago to today; Figure S4. Total number of ROH and total length of ROH segments per individual for each sheep breed; Figure S5. The mean sum of ROH length in Mb per individual for each sheep breed within each ROH length category; Figure S6. Number of ROH per chromosome in the 20 sheep breeds. Table S1. Summary of samples used in this study; Table S2. The candidate genes for European high fecundity trait in the ROH islands in FIN and GOT sheep; Table S3. The candidate genes for Asian high fecundity trait in the ROH islands in HUS, WDS, and SXW sheep; Table S4. The candidate genes for the wool-related trait in the ROH islands in MFW and MSF sheep; Table S5. The candidate genes for the body size trait in the ROH islands in OUE sheep; Table S6. The enrichment analysis results obtained using Panther.
