# Supplementary material for: Genome-Wide Runs of Homozygosity Reveal Inbreeding Levels and Trait-Associated Candidate Genes in Diverse Sheep Breeds
Source: Genes (Basel). 2025 Mar 7;16(3):316. doi: 10.3390/genes16030316 (PMC11942120; doi:10.3390/genes16030316)
Supplement: Supplementary file 1 [file genes-16-00316-s001.zip › Supplementary Figure S1-S6.pdf]

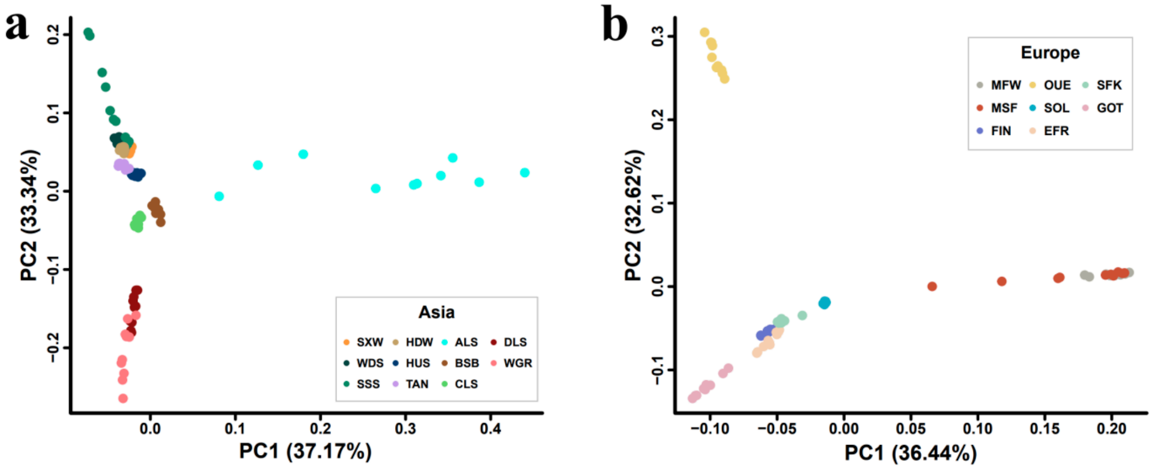

Figure S1. Principal component analysis for Asian and European sheep breeds. (a) Principal component analysis for Asian sheep breeds. (b) Principal component analysis for European sheep breeds.

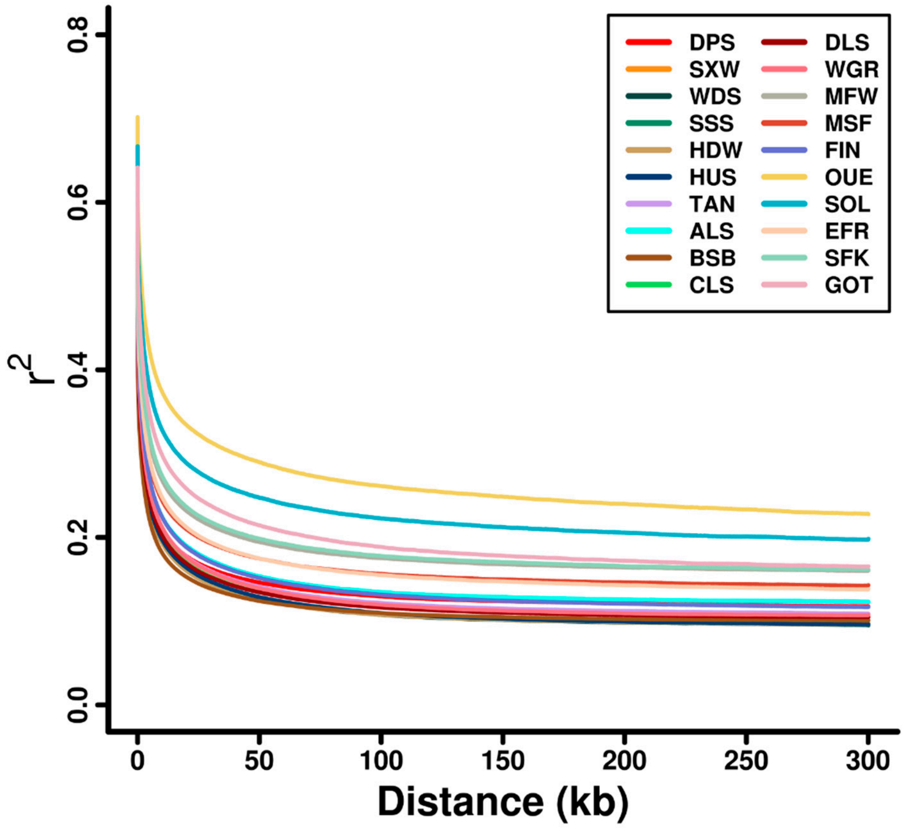

Figure S2. LD decay across genomic distance in the 20 sheep breeds.

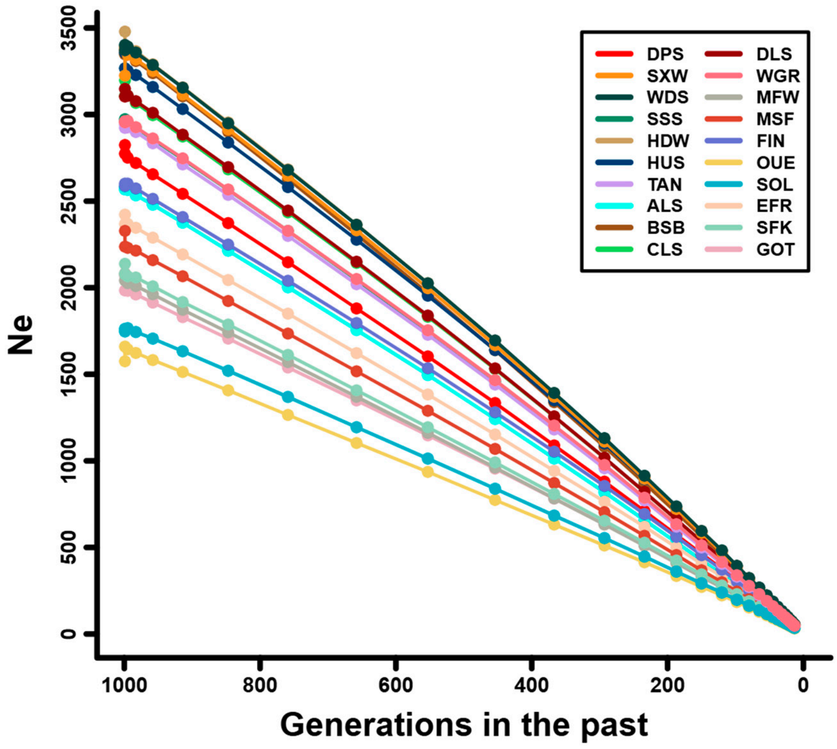

Figure S3. Estimates of Ne for the 20 sheep breeds (or their ancestral populations) from 1000 years ago to today.

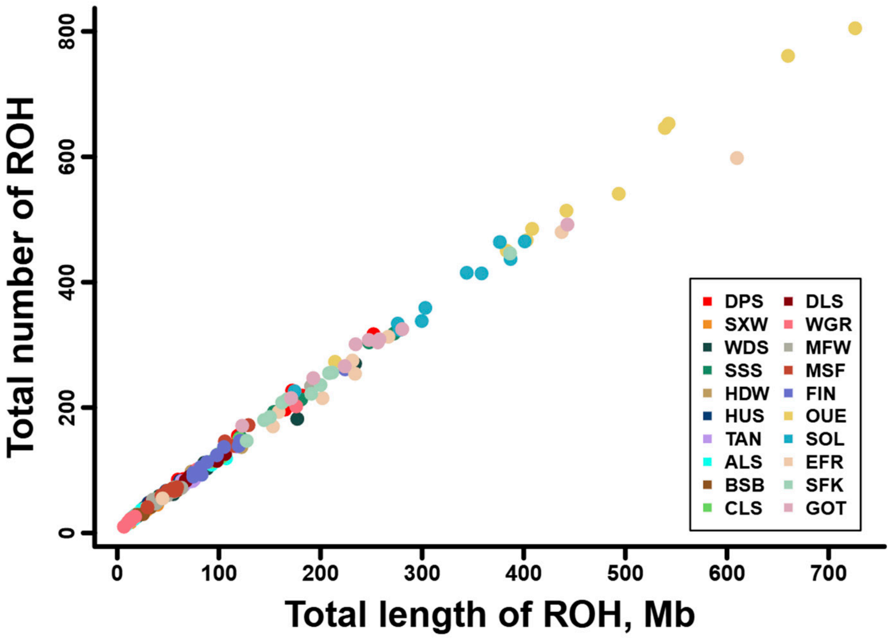

Figure S4. Total number of ROHs and total length of ROH segments per individual for each sheep breed.

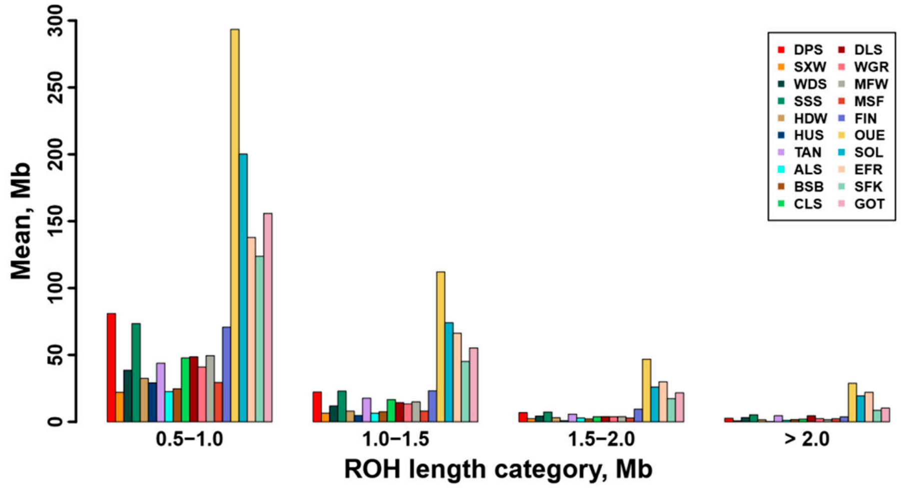

Figure S5. The mean sum of ROH length in Mb per individual for each sheep breed within each ROH length category.

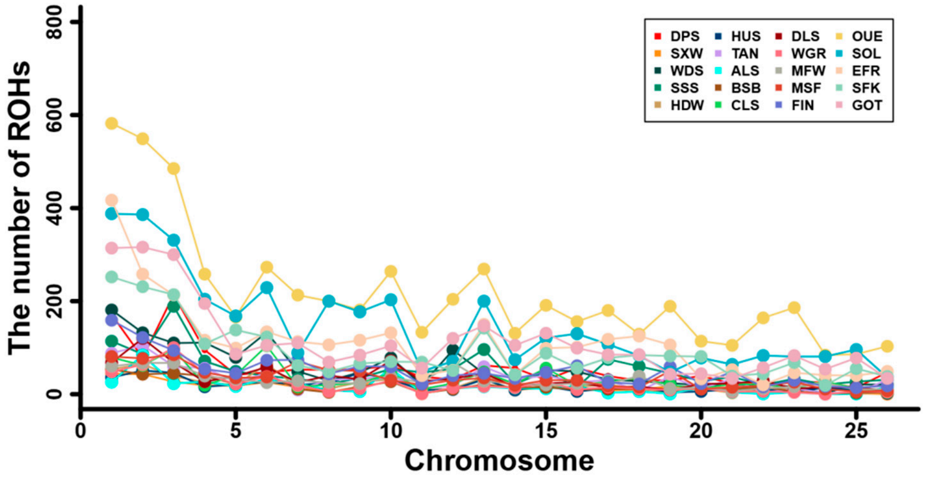

Figure S6. Number of ROH per chromosome in the 20 sheep breeds.
